# An Unusual Case of Hypoglycemia in a Non-diabetic Individual due to Hirata Disease

**DOI:** 10.7759/cureus.56297

**Published:** 2024-03-16

**Authors:** Krishna Padarabinda Tripathy, Debasis Pathi, Pradip Kumar Behera, Sambit Das, Sangam Tarun Venkat Mahesh

**Affiliations:** 1 General Medicine, Kalinga Institute of Medical Sciences, Bhubaneswar, IND; 2 Endocrinology, Kalinga Institute of Medical Sciences, Bhubaneswar, IND

**Keywords:** insulin autoantibodies, autoimmune hypoglycemia, hyperinsulinemic hypoglycemia, insulin autoimmune syndrome, hirata's disease

## Abstract

Hypoglycemia is common in diabetic populations using insulin or insulin secretagogues, but rare in non-diabetics. A 60-year-old non-diabetic male presented with repeated episodes of abnormal behavior persisting for 10-15 minutes for seven days, associated with sweating, intense hunger, and relief on food intake, with no history of insulin or secretagogue intake, with stable vitals and normal systemic examination. Laboratory tests during attacks revealed low blood sugar, high serum insulin, and normal C-peptide levels, with no evidence of pancreatic or extrapancreatic hyperinsulinism, and serum anti-insulin antibody levels >100 U/ml. Based on these results, he was diagnosed with autoimmune insulin syndrome (AIS). Treatment with low-carb meals, oral prednisolone, and acarbose led to the resolution of symptoms. Hirata syndrome, though rare in India, requires consideration as a differential diagnosis to avoid unnecessary invasive procedures.

## Introduction

Hypoglycaemia is common in the diabetic population on insulin and secretagogues, however, hypoglycemia in people without diabetes is rare. Hypoglycaemia is a medical condition characterized by a decrease in the concentration of glucose (sugar) in the bloodstream below normal levels. The American Diabetes Association and the International Hypoglycaemia Study Group define clinically significant hypoglycemia as a blood glucose <54 mg/dl (3.0 mmol/L), which is detected by an individual’s self-monitoring blood glucose (SMBG) as well as by continuous glucose monitoring (CGM) [1,2].

Hypoglycaemia can be documented by Whipple's triad: (1) symptoms consistent with hypoglycemia, (2) low plasma glucose measured with a precise method, and (3) the relief of symptoms after plasma glucose is raised [3].

Evaluation of hypoglycaemia in an individual with diabetes mellitus revolves around excessive dosage of insulin or insulin secretagogue, organ failure, infections, and skipping of meals, however, hypoglycaemia is rare in individuals without diabetes mellitus and it can be insulin-mediated (hyperinsulinism) or non-insulin-dependent. Endogenous insulin-mediated hypoglycaemia in an adult without diabetes mellitus can be secondary to insulinoma, islet cell hyperplasia (nesidioblastosis), Type B insulin resistance, and iatrogenic hyperinsulinism causing hypoglycaemia which can be due to exogenous insulin intake or insulin secretagogues [4].

Insulin-independent hypoglycemia encompasses various conditions, including alcohol consumption, visceral failure (involving the liver or kidneys), critical illness, primary adrenal failure, anterior pituitary failure, severe sepsis, cerebral malaria, anorexia nervosa, glycogen storage disease, post-bariatric surgery, mesenchymal tumours with elevated IGF-2 levels, autoimmune hypoglycemia due to anti-insulin antibodies or anti-insulin receptor antibodies, and certain medications [4].

Causes of fasting hypoglycaemia are usually inherited liver enzyme deficiencies, inherited defects in fatty acid oxidation, non-insulinoma pancreatogenous hypoglycaemia syndrome (NIPHS), post-bariatric surgery hypoglycaemia, and hereditary fructose intolerance [5].

## Case presentation

A 60-year-old male farmer presented with complaints of abnormal behaviour, confusion and head reeling for seven days which were sudden in onset. The patient's attendants noticed that he experienced symptoms in the early hours of the morning and that symptoms were normalized after consuming sugar water or carbohydrate-rich meals.

The patient had no history of diabetes mellitus, no history of exposure to insulin or insulin secretagogues, or any drug known to precipitate hypoglycemia. There was no family history of diabetes or insulinoma. He was diagnosed with hypertension a few days before admission and was started on telmisartan tablets (40 mg) + amlodipine (5mg).

On admission, the patient's vitals were stable, a systemic examination was normal, and there were no external markers of insulin resistance. He was found to be in a confused state with abnormal behaviour; during this event, capillary blood sugar was 37 mg/dl; however, his other vitals were normal. The patient immediately improved after injecting a 25% dextrose solution. The patient had similar episodes during the subsequent days of his hospital stay.

While evaluating the cause of fasting hypoglycemia, the critical sample at the time of the hypoglycemic episode was (Table 1).

**Table 1:** Lab Parameters CLIA: chemiluminescent immunoassay; ELISA: enzyme-linked immunosorbent assay; HPLC: high-performance liquid chromatography; EIA: enzyme immunoassay

Parameter	Value	Reference range	Method
Critical blood sugar	24 mg/dl	nil	Hexokinase
Fasting blood sugar	93 mg/dl	70-100 mg/dl	Hexokinase
Serum insulin	240.38 µIU/mL	1.9 – 23.0 µIU/mL	CLIA
Serum C peptide	6.40ng/ml	0.05-30.0 ng/ml	ELISA
HbA1c	5.9 %	Non-diabetic: <5.7; pre-diabetic – 5.7 -6.4; diabetic: 6.5	HPLC
Blood ketone (beta-hydroxybutyrate)	0.04 mg/dl	0.21-2.81 mg/dl	ELISA
S. Anti-insulin antibody	>100 U/ml	<10 U/ml	EIA
S. Cortisol (4 pm)	20.09 mcg/dl	<10 mcg/dl	CLIA
S. Cortisol (8 am)	28.89 mcg/dl	6.7-22.6 mcg/dl	CLIA
Growth hormone	0.383 ng/ml	0.030-2.47 ng/ml	ECLIA

Liver, kidney and thyroid function tests were within normal ranges (Table 2). 

**Table 2:** Liver, kidney and thyroid function tests SGOT: serum glutamic oxaloacetic transaminase; SGPT: serum glutamic pyruvic transaminase; ALP: alkaline phosphatase; GGT: gamma-glutamyl transferase; TSH: thyroid stimulating hormone

Lab parameters	Value	Normal range
Serum bilirubin	0.26 mg/dl	0.2-1.2 mg/dl
Serum SGOT	29 U/L	0 - 40 U/L
Serum SGPT	30 U/L	5 - 40 U/L
Serum ALP	101 U/L	40-129 U/L
Serum GGT	22 U/L	10-60 U/L
Serum albumin	4.3 gm/dl	3.5-5.0 gm/dl
Serum urea	17 mg/dl	12-42 mg/dl
Serum creatinine	1.11 mg/dl	0.7-1.3 mg/dl
Serum sodium	137 mmol/L	136-146 mmol/L
Serum potassium	3.7 mmol/L	3.5-5.1 mmol/L
Serum calcium	8.8 mg/dl	8.6-10.3 U/L
Serum uric acid	5.1 mg/dl	3.4-7.0 mg/dl
FT4	0.91 ng/dl	0.5-1.5 ng/dl
TSH	0.893 micro IU/ml	0.35-4.2 micro IU/ml

Due to the presence of recurrent hypoglycemic attacks, the patient was evaluated. As serum insulin levels were high, he was evaluated for hyperinsulinemic hypoglycemia. Detailed history-taking confirmed no intake of insulin secretagogues or prior insulin exposure proved by normal C-peptide levels; so to rule out other causes of endogenous insulin secretion, further investigations were done.

Endoscopic ultrasonography (EUS) and abdominal computed tomography (CECT abdomen) with contrast showed no pancreatic or extra-pancreatic lesions. Ga68 DOTANOC PET-CT was performed and there was no abnormal focus of somatostatin receptor uptake in the whole body. No evidence of abnormal somatostatin receptor-expressing lesions was found in the rest of the body.

Antinuclear antibody test (ANA) profile and serum protein electrophoresis showed no abnormality, and serology for viral hepatitis was negative. As the insulin to C-peptide ratio is >1, serum insulin antibody titers were done with high suspicion of insulin antibody syndrome and the antibody titers were higher >100, so with findings of hypoglycemic episodes, high serum insulin levels, normal C-peptide levels, high titers of serum insulin antibody, there was no evidence of pancreatic or extrapancreatic insulin release. The patient was diagnosed with autoimmune insulin syndrome.

The patient was started on small and frequent low-carbohydrate meals, and oral prednisolone was started at 60 mg per day (1mg/kg/day) with acarbose tablets. The patient improved symptomatically and was discharged. On follow-up after 60 days, the patient improved and there were no subsequent hypoglycemic episodes. Blood sugars were within normal range, with fasting blood sugar of 85 mg/dl and post-prandial blood sugar test (PPBS) of 128 mg/dl, and oral steroid (prednisolone) was tapered to a dose of 20 mg/day.

## Discussion

Autoimmune insulin syndrome(AIS) may occur with fasting or exercise hypoglycaemia; however, it is classically characterized by late postprandial hypoglycaemia, high insulin levels, and positive results for anti-insulin antibodies. Insulin to C-peptide molar ratio has been proposed as a diagnostic tool for AIS [6].

Autoimmune insulin syndrome is the third leading cause of hypoglycemia in the Japanese population with around 380 reported cases. AIS seems to affect both genders equally [6], but this condition is rare among Indians, only 28 cases are reported in India. Usually reported in the seventh decade of life, however, nowadays age at onset varies [7].

AIS has been described as a type VII hypersensitivity. Insulin autoantibodies are immunoglobulins directed against insulin molecules; they are more commonly IgG, glycated haemoglobin can be normal or increased and may vary according to the frequency and severity of hypoglycemia.

Type B insulin resistance is another form of autoimmune hypoglycemia due to the presence of autoantibodies that bind to insulin receptors, which exert an agonistic effect resulting in insulin resistance and hypoglycemia (paradoxical). These patients have severe insulin resistance presenting as severe diabetes mellitus poorly responsive to insulin therapy. The reliable method for differentiating these two types of autoimmune hypoglycemia is by testing for the presence of autoantibodies in AIS and anti-receptor insulin antibodies in Type B insulin resistance [8].

**Figure 1:**
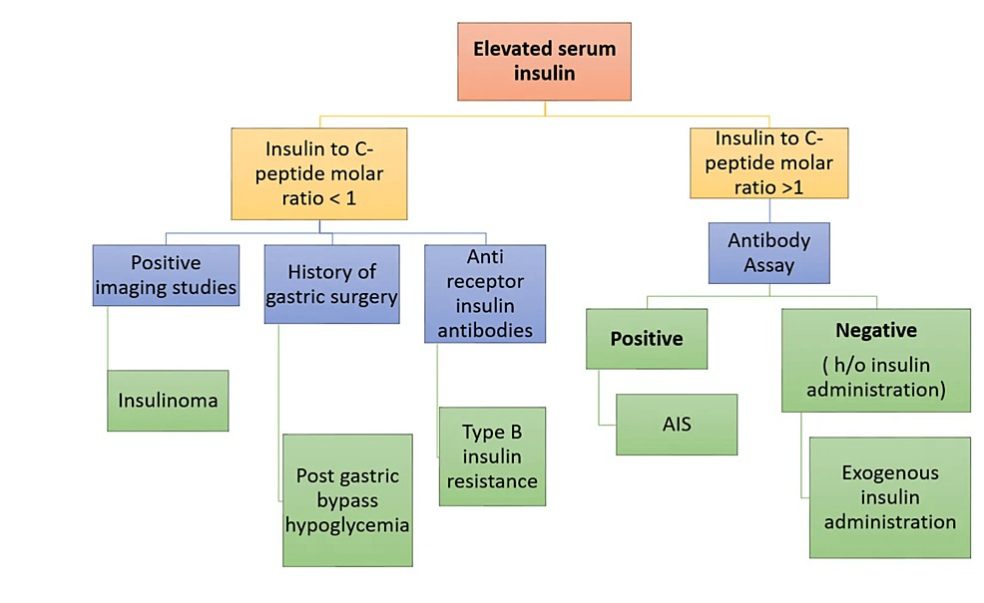
Flowchart depicting a diagnostic approach AIS: autoimmune insulin syndrome

AIS patients usually present with high insulin concentrations, which is a rare finding in other forms of hyperinsulinemia hypoglycemia. C-peptide and proinsulin are useful in differentiating endogenous and exogenous insulin secretion. Imaging studies will be normal in AIS. In AIS, as a result of IAA bound to insulin, the insulin t1\2 increases from five minutes to hours, while the half-life of C-peptide usually remains unaffected (30-35 minutes). Patients with IAS thus typically have high serum insulin concentrations, while their C-peptide and proinsulin levels may or may not be raised (Figure 1) [9].

The genetic association was found in the Japanese population with AIS, human leukocyte antigen (HLA)-DR4 (48 out of 50 patients vs. controls; odds ratio 39.6). HLA-DRB1*0406 is quite prevalent among Eastern Asian patients, whereas Caucasian AIS patients mainly express HLA-DRB1*0403, and have a low prevalence of DRB1*0406 [10].

It was estimated that drug-induced AIS accounted for about 50% of the total in the Caucasian population. AIS has a high strength of association with anti-thyroid drugs and alpha lipoic acid, while other drugs have a low strength of association (less than five case reports) [11]; notably, in a recent study, a case report was reported with AIS in a male patient taking alpha-lipoic acid [12].

The pharmacological therapy of AIS includes glucocorticoids, somatostatin analogues, diazoxide, azathioprine and rituximab, approximately 82% of the AIS patients underwent spontaneous remission in a study of 197 patients diagnosed with IAS from 1970 to 1992 [13]. So rather than pharmacological therapy, dietary modification achieved good results when small, frequent low-carbohydrate meals were advised; this prevented postprandial hyperglycemia causing insulin secretion and further precipitating hypoglycemic attacks [11].

Uncooked cornstarch, acarbose (a alpha-glucosidase inhibitor) also showed good results in some subset of people. Metformin has also been tried in some studies as it reduces insulin resistance, thereby decreasing insulin-insulin autoantibody complexes. The recurrence of AIS following full resolution is low; in a Japanese study of 197 patients only nine patients showed recurrence, and it does not have any trigger factor [11].

## Conclusions

Autoimmune insulin syndrome is rare but an important most overlooked cause of hypoglycaemia, which requires detailed assessment for diagnosis. It may be associated with different autoimmune diseases as it is a self-limiting disease and can be diagnosed by testing for high serum insulin levels, insulin/c-peptide molar ratio of more than one, and the presence of insulin autoantibodies.

So once the diagnosis is confirmed, the patient must be carefully assessed and should be started on pharmacological therapy. Usually, AIS is self-remitting and treatment with diet modification and immunosuppressants are often helpful. Suspecting this rare entity is crucial as it will spare the patient from avoidable aggressive investigations and unwarranted surgical procedures.
